# Risk factors of recurrent periprosthetic joint infection of the knee after two-stage reimplantation

**DOI:** 10.1186/s43019-025-00258-5

**Published:** 2025-01-14

**Authors:** Do Weon Lee, Hyuk-Soo Han, Du Hyun Ro

**Affiliations:** 1 Department of Orthopedic Surgery, Dongguk University Ilsan Hospital, Goyang, Gyunggi-Do South Korea; 2 Department of Orthopedics, Seoul National University College of Medicine, Seoul, South Korea; 3 Department of Orthopedic Surgery, Seoul National University Hospital, 101 Daehak-Ro, Jongno-Gu, Seoul, 110-744 South Korea; 4 Innovative Medical Technology Research Institute, Seoul National University Hospital, Seoul, South Korea; 5 5CONNECTEVE Co., Ltd, Seoul, South Korea

**Keywords:** Total knee arthroplasty, Prosthesis-related infection, Survival analysis

## Abstract

**Introduction:**

Prosthetic joint infection (PJI) is one of the most common and detrimental complications of total knee replacement arthroplasty (TKA). Despite extensive efforts, including two-stage reimplantation, to eradicate PJI, it still recurs in a substantial number of patients. However, the risk factors of recurrence after two-stage reimplantation of the knee have not been established. In this study, it is hypothesized that there will be certain risk factors of recurrence after two-stage reimplantation for PJI of the knee.

**Materials and methods:**

From March 2002 to December 2022, 65 knees that underwent two-stage reimplantation for PJIs in a single, tertiary hospital were retrospectively reviewed, and 44 patient-related, laboratory-related, and surgery-related factors, including body mass index, pathogen type, and the usage of transfusions, were selected as the potential risk factors for recurrence. Survival analysis using the Kaplan–Meier method and subsequent Cox proportional hazard regression were performed.

**Results:**

Out of the 65 knees that underwent two-stage reimplantation, infection recurred in 15 knees (23.1%) in a median 11 (range 4–108) months. The Cox proportional hazards regression showed that infection of revision TKA, mixed pathogen-type infection, and higher serum erythrocyte sedimentation rate (ESR, mm/h) level increases the risk of recurrence (*p*-values < 0.001, 0.04, and 0.009; hazard ratios 40.29, 1.53, and 1.03, respectively).

**Conclusions:**

A significant portion of PJI of the knees recurred after two-stage reimplantation. Revision TKA at the time of initial PJI, mixed pathogen-type infection, and higher serum ESR level were three significant risk factors of PJI recurrence. Surgeons should be more cautious in suspecting PJI relapse for these specific occasions.

**Level of evidence:**

III, retrospective cohort study.

## Introduction

Prosthetic joint infection (PJI) is one of the most common and detrimental complications of total knee replacement arthroplasty (TKA), occurring in about 1–2% of cases [1, 2]. Except for the cases of acute PJI after TKA or acute hematogenous PJI [3], the current gold standard treatment for PJI of the knee is a two-staged surgery in which antibiotics-laden cement is first inserted, and prosthesis is reimplanted after certain period of time, during which systemic antibiotics are first used, and then stopped (antibiotic-free period) [4]. The duration of the antibiotics and the antibiotic-free period varies according to the type of pathogen that caused PJI [5].

Despite these extensive efforts in eradicating PJI of the knee, PJI still recurs in a substantial number of patients, requiring several more surgeries and subsequently causing significant economic burden and sequelae [6, 7]. Moreover, according to recent publications [8, 9], repeat two-stage revision for knee PJI has a very high failure rate, which accentuates the importance of infection control in the first two-stage revision surgery. The risk factors of PJI in initial TKA are relatively well known, including several patient factors (male sex, diabetes mellitus, rheumatoid arthritis, and preoperative nutritional status) [10] and surgery-related factors (revision TKA and use of blood transfusion) [11]. However, the risk factors of PJI recurrence after two-stage reimplantation of the knee have not been yet established, despite some recent literature on the topic [12, 13].

The purpose of this study was to identify the risk factors for PJI recurrence following two-stage reimplantation by performing a survival analysis. Using the Cox proportional hazards model, we aimed to determine which factors are associated with an increased risk of recurrence in a time-dependent manner. The authors hypothesized that certain patient- or treatment-related factors would be significant predictors of these recurrences.

## Patients and methods

**Fig. 1:**
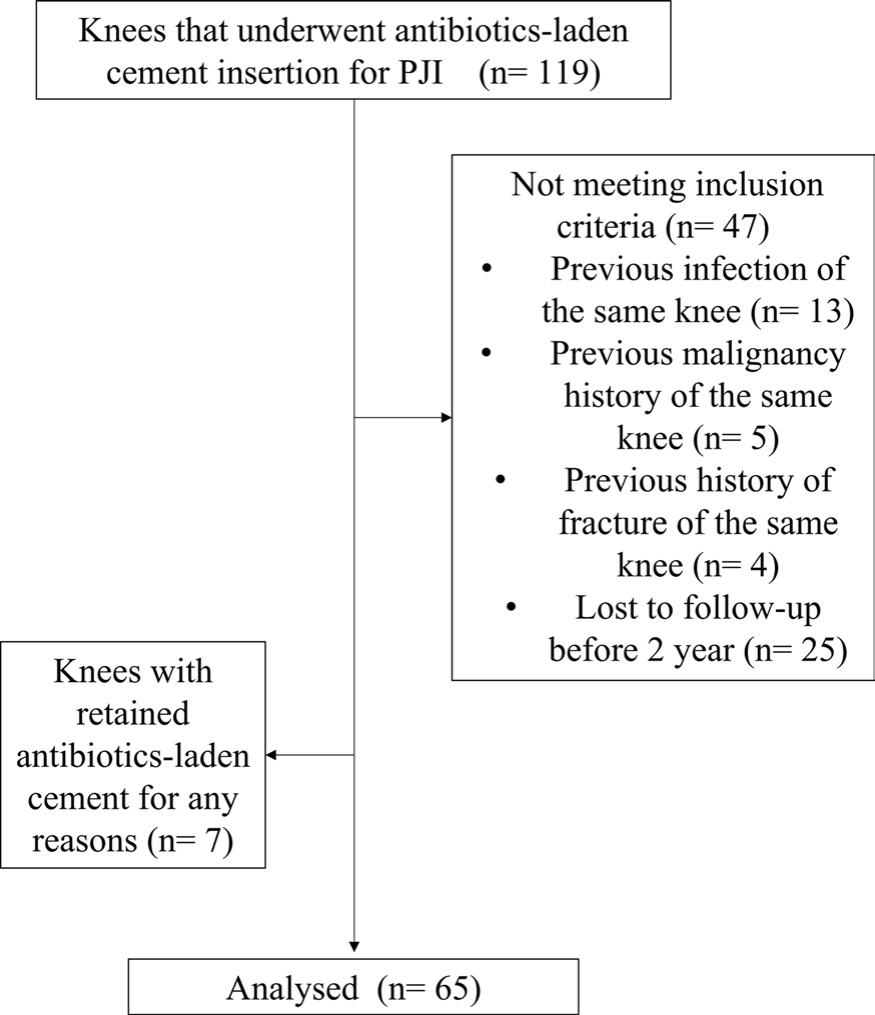
Flow chart of patient selection in the study

This retrospective cohort study was approved by the institutional review board of the hospital (no. H. 0812-019-265). From March 2002 to December 2022, 119 consecutive knees that underwent antibiotics-laden cement spacer insertion for prosthetic joint infection (PJI) in a single, tertiary hospital were investigated. Out of these patients, patients who had prior history of infection (*n* = 13), malignancy (*n* = 5), or fracture (*n* = 4) on the same knee; patients who had retained cement spacer without prosthesis reimplantation (*n* = 7); and patients who were lost to follow-up before 2 years (*n* = 25) were excluded (Fig. 1). After the exclusions, a total of 65 knees were selected for assessment. All knees were followed up on for at least 2 years after reimplantation. The knees in which the debridement, antibiotics, and implant retention (DAIR) procedure was performed prior to the insertion of antibiotics-laden cement spacers were not excluded because the authors believed that this would better reflect a real-world scenario and avoid selection bias. Therefore, referred cases of PJI from other hospitals were included in this study, and prior DAIR procedure was analyzed as an independent risk factor in the study.

Initial PJI of the knee was diagnosed using the 2018 Musculoskeletal Infection Society (MSIS) criteria [14]. Subsequently, adequate systemic and local antibiotics for the cement spacer were selected with the consultation of infectious disease specialists. Systemic antibiotics were used for 6 weeks, with the following 6 weeks being an antibiotic-free period, except for the cases of fungal PJI. Fungal PJIs were managed with a longer time of antifungal usage and a longer duration of antifungal-free period (generally, 3 months each), which was decided with careful discussion with the infectious disease specialists. Reimplantation was performed when infection eradication was confirmed by a sterile joint aspiration culture, a low polymorphonuclear count in frozen biopsy tissue (< 5 PMNs per high-power field), and normalized ESR and CRP levels, along with the absence of clinical signs of infection. These criteria were evaluated after a 6-week antibiotic-free period (or longer for fungal PJI). If eradication was not confirmed, reimplantation was delayed, and the cement spacer was exchanged. After reimplantation, antibiotics were not used for an extended period in any of the cases (less than 1 week). PJI recurrence after two-stage reimplantation was diagnosed using the 2018 MSIS criteria.

### Clinical variables

Demographic factors including age (years), sex, height (cm), body mass index (BMI, kg/m^2^), 18 patient factors (American Society of Anesthesiologists [ASA] status, hypertension, diabetes mellitus, rheumatoid arthritis of the affected knee, depression, cardiovascular disease other than hypertension, cerebrovascular disease, dementia, history of malignancy, chronic kidney disease, chronic liver disease, chronic pulmonary disease, neurological disease, peripheral vascular disease, other rheumatic diseases, active steroid usage, smoking, and alcohol abuse), 6 laboratory factors (erythrocyte sedimentation rate [ESR, mm/hr], C-reactive protein [CRP, mg/dL], hemoglobin [g/dL], albumin [g/dL], prognostic nutritional index [PNI, calculated as serum albumin {g/L} + 5 × total lymphocyte count {10^9^/L}] [15] and synovial fluid white blood cell count [cells/µL]) and 16 surgical factors (effective surgical time, tourniquet time, estimated blood loss, whether bone augments were used for significant bone defects, whether extensive surgical approach including V–Y turndown or rectus femoris muscle snip was applied, whether hinged-type implant was used due to irreparable ligamentous instability, pathogen type [classified into 4 groups; Gram positive {G( +)}, Gram negative {G(−)}, fungus, and mixed], presence of antibiotic resistance [e.g., Methicillin-resistant *Staphylococcus aureus* {MRSA}], culture positivity, presence of sinus tract, usage of transfusions, total number of cement spacer insertion surgeries prior to prosthesis reimplantation, whether the initial infection was primary TKA or revision TKA, whether DAIR procedure was performed prior to first cement spacer insertion, time from initial surgery to cement spacer insertion, and time from cement spacer insertion to prosthesis reimplantation) were evaluated (a total of 44 factors). The factors were chosen considering the known risk factors of PJI of TKA according to previous literature [10, 11]. For revision TKA PJIs, only aseptic revision cases were included after thorough clinical review.

First, the two groups of knees (knees in which PJI recurred versus knees in which PJI did not recur) were compared. Second, a survival analysis was performed with the endpoint being the recurrence of PJI. Subsequently, the Cox proportional hazards regression model was performed using the above-mentioned 44 factors to find the statistically significant factors for PJI relapse. Afterward, a survival curve was separately drawn for each of the statistically significant variables in the Cox proportional hazards model, and they were compared between the groups. Lastly, as an ancillary analysis, the knees in which infection recurred with the same type of pathogen as the initial PJI were compared with those in which infection recurred with a different type of pathogen to identify whether there were any differences in patient- or surgery-related factors.

### Statistical analysis

All of the statistical analyses were performed using the RStudio version 2022.07.01 (RStudio, PBC, Boston, MA URL http://www.rstudio.com/). Continuous variables are presented as means and standard deviations, while categorical variables are presented as frequencies and percentages. The survival analysis was performed using the Kaplan–Meier method, and a Cox proportional hazards regression was subsequently performed. The comparison between the groups (no relapse versus relapse and relapse by same pathogen versus relapse by different pathogen) were performed with the student *t*-test and Fisher’s exact test for continuous and categorical variables, respectively. Both the Shapiro–Wilk test and Levene’s test were performed, confirming the normality and equal variances of all data. A *p*-value below 0.05 was considered statistically significant.

The demographics of the study patients are presented in Table 1.

**Table 1:** Demographics of the patients in the study

	Relapse group	Non-relapse group	*p*-Value
No. of knees	15	50	
Age (years)	73.0 ± 6.5	70.4 ± 8.5	0.220
Sex (male/female)	3/12	5/45	0.373
Height (cm)	157.0 ± 8.7	153.6 ± 7.1	0.177
BMI (kg/m^2^)	25.9 ± 3.8	26.0 ± 4.4	0.931

Relapse group, knees with recurred prosthetic joint infection; non-relapse group, knees without recurrence of prosthetic joint infection; BMI, body mass index

## Results

The average length of follow-up after two-stage reimplantation was 4.8 years. The minimum and average follow-up period for the non-relapse group were 2 and 5.6 (range 2–16) years, respectively. Of the 65 knees that underwent antibiotics-laden cement spacer insertion for PJI of the knee, infection recurred in 15 knees (23.1%). When the clinical factors were compared between the knees in which infection recurred and the knees in which infection did not recur, there were two significant factors that differed between the groups (Table 2). Higher level of serum ESR (mm/h) and revision-type TKA were significantly associated with PJI recurrence (*p* = 0.002 and *p* < 0.001, respectively). A total of 12 out of 15 knees (80.0%) in the recurrence group were PJIs on revision TKAs, while only 2 out of 50 knees (4.0%) in the non-recurrence group were PJIs on revision TKAs.

**Table 2:** Comparison of the clinical factors between the two groups (relapse versus no-relapse)

	Relapse group (*n* = 15)	Non-relapse group (*n* = 50)	*p*-Value
*Patient factors*			
ASA status			
1	1	9	0.412
2	14	38	
3	0	3	
Hypertension (yes/no)	12/3	26/24	0.075
Diabetes mellitus (yes/no)	6/9	16/34	0.792
Rheumatoid arthritis of the affected knee (yes/no)	1/14	2/48	0.551
Cardiovascular disease (yes/no)	1/14	4/46	1.000
Cerebrovascular disease (yes/no)	1/14	4/46	1.000
Depression (yes/no)	0/15	3/47	1.000
Dementia (yes/no)	0/15	2/48	1.000
History of malignancy (yes/no)	1/14	4/46	1.000
Chronic kidney disease (yes/no)	1/14	2/48	1.000
Chronic liver disease (yes/no)	0/15	1/49	1.000
Chronic pulmonary disease (yes/no)	1/14	1/49	0.411
Neurological disease (yes/no)	0/15	3/47	1.000
Peripheral vascular disease (yes/no)	0/15	0/50	1.000
Other rheumatic disease (yes/no)	0/15	1/49	1.000
Active steroid usage (yes/no)	0/15	1/49	1.000
Smoking (yes/no)	0/15	2/48	1.000
Alcohol abuse (yes/no)	2/13	1/49	0.131
*Laboratory factors*			
Hemoglobin (g/dL)	11.4 ± 1.4	11.0 ± 1.2	0.347
Albumin (g/dL)	3.5 ± 0.5	3.7 ± 0.5	0.272
PNI	57.6 ± 13.0	62.2 ± 10.6	0.244
CRP (mg/dL)	6.8 ± 9.1	4.6 ± 4.1	0.464
ESR (mm/h)	98.3 ± 20.3	68.7 ± 18.6	**0.002**
Synovial WBC count (*1000 cells/ µL)	28.6 ± 24.7	23.2 ± 17.3	0.616
*Surgical factors*			
Effective surgical time (min)	176.9 ± 43.6	182.6 ± 42.6	0.684
Tourniquet time (min)	125.9 ± 29.8	120.5 ± 32.9	0.581
Estimated blood loss (ml)	448.5 ± 297.9	524.2 ± 426.5	0.469
Extensive surgical approach (yes/no)	1/14	6/44	1.000
Hinged implant due to irreparable ligamentous instability (yes/no)	2/13	3/47	0.273
Significant bone defects requiring augments (yes/no)	13/2	48/2	0.506
Pathogen type			
G( +)	10	42	0.167
G(−)	1	2	
Fungus	2	5	
Mixed	2	1	
Antibiotics resistance (yes/no)	6/9	19/29	1.000
Positive culture (yes/no)	15/0	48/2	1.000
Sinus tract (yes/no)	3/12	16/34	0.522
Usage of transfusions	2/13	3/47	0.325
Total number of cement spacer insertion			
1	10	44	0.083
2	5	5	
3	0	1	
TKA type (primary/revision)	3/12	48/2	**< 0.001**
DAIR surgery prior to cement spacer insertion	6/9	21/29	1.000
Time from initial surgery to cement spacer insertion (months)	45.4 ± 46.3	49.7 ± 72.0	0.789
Time from cement spacer insertion to prosthesis reimplantation (months)	4.4 ± 2.8	5.1 ± 5.0	0.493

Relapse group, knees with recurred prosthetic joint infection; non-relapse group, knees without recurrence of prosthetic joint infection; ASA, American Society of Anesthesiologists; PNI, prognostic nutritional index; CRP, C-reactive protein; ESR, erythrocyte sedimentation rate; WBC, white blood cell; G( +), Gram stain positive; G(−), Gram stain negative; TKA, total knee replacement arthroplasty; DAIR, debridement, antibiotics, and implant retention. *p*-Value below 0.05 is written in bold text to note statistical significance

**Fig. 2:**
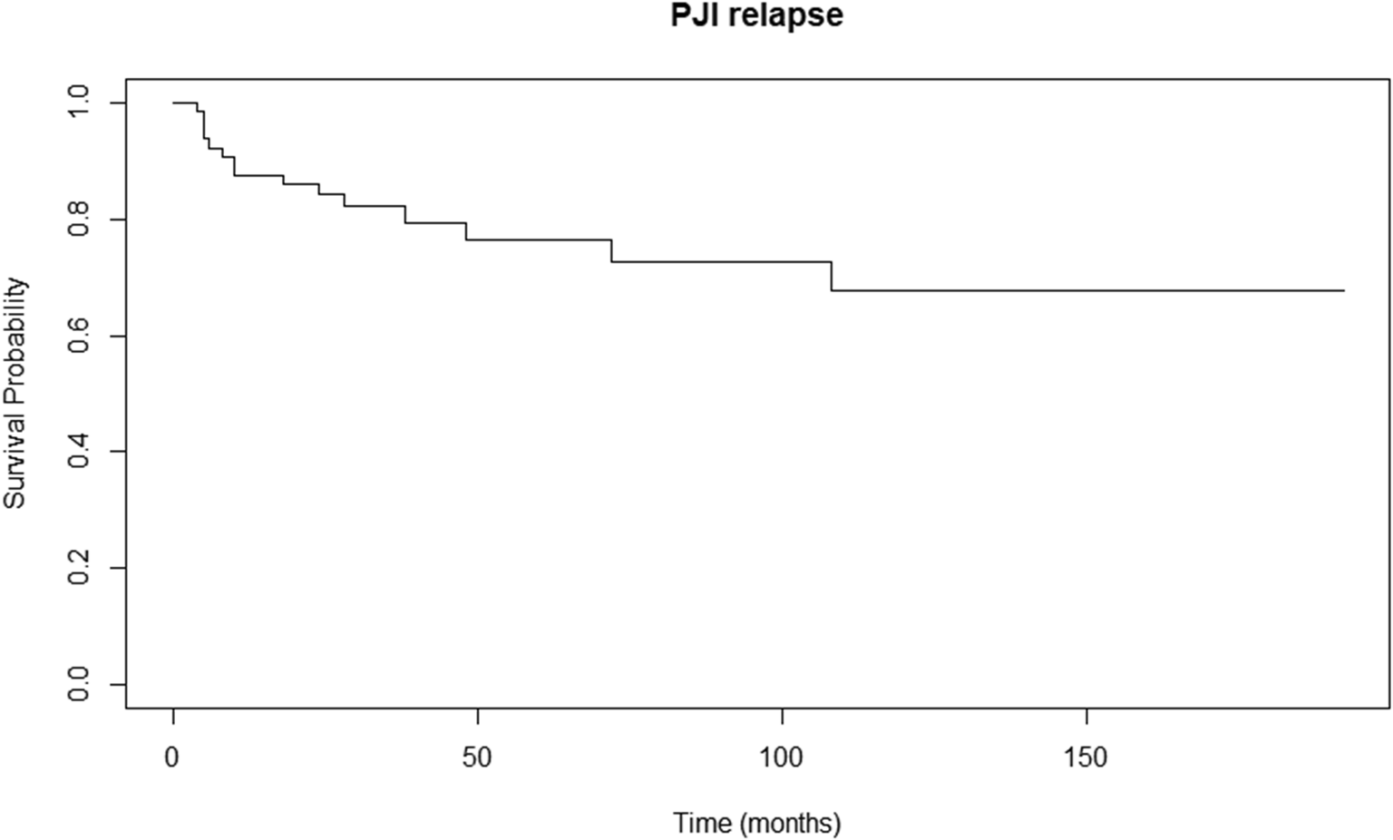
PJI relapse survival curve of the study patients (whole). PJI, prosthetic-joint infection

**Fig. 3:**
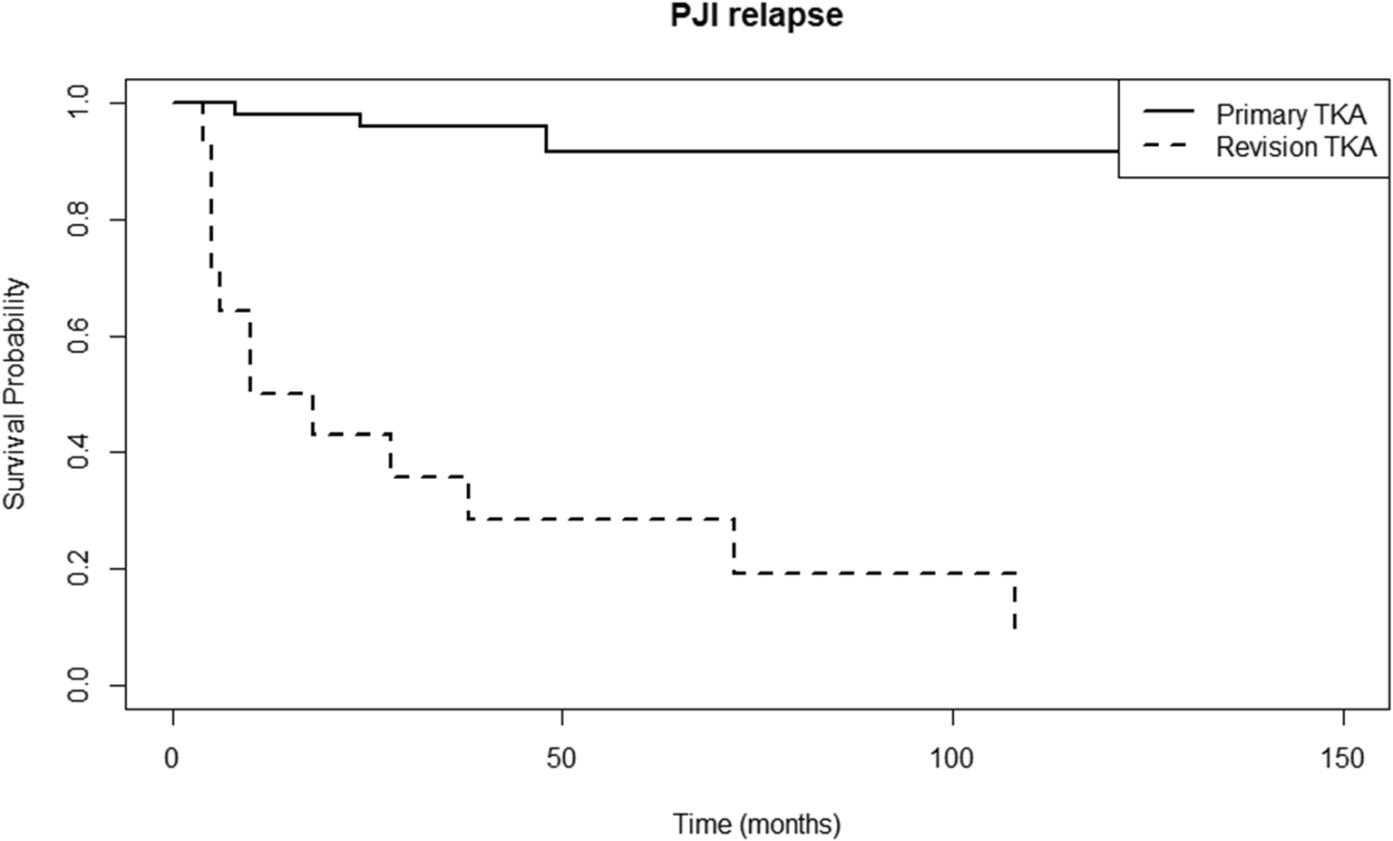
PJI relapse survival curve of the patients according to the type of surgery before the initial prosthetic joint infection (primary TKA versus revision TKA). PJI, prosthetic joint infection; TKA, total knee replacement arthroplasty

**Fig. 4:**
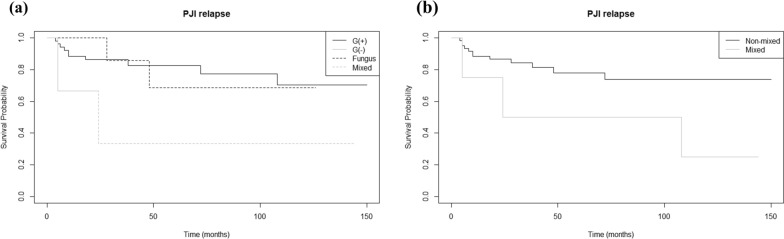
PJI relapse survival curve of the patients according to the type of pathogen that caused the initial PJI; comparison between all four pathogen types (**a**) and non-mixed pathogen type versus mixed pathogen type (**b**). PJI, prosthetic joint infection; G( +), Gram positive; G(−), Gram negative; non-mixed, non-mixed pathogen type; mixed, mixed pathogen type

The survival analysis curve of the whole group of study patients is shown in Fig. 2. PJI recurred in 15 out of 65 knees and the median time to recurrence was 10 (range 4–108) months. Subsequent Cox proportional hazards regression resulted in three statistically significant factors: type of TKA at initial PJI (either primary or revision), mixed-type pathogen, and ESR level (*p*-values: < 0.001, 0.04, and 0.009, hazard ratio: 40.29, 1.53, and 1.03, respectively). The survival curves for different types of TKA and pathogens are shown in Figs. 3 and 4, respectively. Revision TKA at the time of initial PJI and mixed pathogen-type infection showed higher probability of PJI recurrence over the follow-up period. Each of the three mixed pathogen-type infections in our study were combinations of G(+) and G(−) bacteria. An optimal ESR cutoff of 75.8 mm/h was identified for predicting event occurrence, using a receiver operating characteristic (ROC) curve analysis, with the threshold determined based on Youden’s Index.

The ancillary analysis that compared the knees in which infection recurred with the same pathogen as the initial PJI (*n* = 4) with the knees in which infection recurred with a different pathogen (*n* = 11) showed that there were no clinical differences between the groups except for serum albumin level and the time from cement spacer insertion to prosthesis reimplantation (Table 3). Time from cement spacer insertion to prosthesis reimplantation was significantly higher in the group that recurred with a different type of pathogen. Although the average time to recurrence was shorter in the knees that recurred with the same pathogen (1.2 versus 2.5 months), it was not statistically significant (*p* = *0.382*).

**Table 3:** Comparison of the clinical factors between the two groups in which relapse occurred (same pathogen relapse versus different pathogen relapse)

	Same pathogen (*n* = 4)	Different pathogen (*n* = 11)	*p*-Value
*Average time to recurrence (months)*	1.2 ± 1.3	2.5 ± 2.8	0.382
*Patient factors*			
Age (years)	75.5 ± 5.7	72.0 ± 6.7	0.358
Sex (male/female)	1/3	2/9	1.000
Height (cm)	158.6 ± 8.0	156.4 ± 9.2	0.661
BMI (kg/m^2^)	26.0 ± 3.3	25.8 ± 4.2	0.950
ASA status			
1	0	1	1.000
2	4	10	
3	0	0	
Hypertension (yes/no)	4/0	8/3	0.517
Diabetes mellitus (yes/no)	1/3	5/6	0.604
Rheumatoid arthritis of the affected knee (yes/no)	1/3	0/11	0.267
Cardiovascular disease (yes/no)	0/4	1/10	1.000
Cerebrovascular disease (yes/no)	0/4	1/10	1.000
Depression (yes/no)	0/4	0/11	1.000
Dementia (yes/no)	0/4	0/11	1.000
History of malignancy (yes/no)	1/3	0/11	0.267
Chronic kidney disease (yes/no)	1/3	0/11	0.267
Chronic liver disease (yes/no)	0/4	0/11	1.000
Chronic pulmonary disease (yes/no)	0/4	1/10	1.000
Neurological disease (yes/no)	0/4	0/11	1.000
Peripheral vascular disease (yes/no)	0/4	0/11	1.000
Other rheumatic disease (yes/no)	0/4	0/11	1.000
Active steroid usage (yes/no)	0/4	0/11	1.000
Smoking (yes/no)	0/4	0/11	1.000
Alcohol abuse (yes/no)	0/4	2/9	1.000
*Laboratory factors*			
Hemoglobin (g/dL)	10.1 ± 1.3	11.8 ± 1.1	0.062
Albumin (g/dL)	3.0 ± 0.1	3.7 ± 0.6	**0.001**
PNI	55.4 ± 1.6	58.2 ± 14.8	0.553
CRP (mg/dL)	5.9 ± 2.4	7.6 ± 11.1	0.688
ESR (mm/h)	100.0 ± 30.0	97.9 ± 22.7	0.919
Synovial WBC count (*1000 cells/ µL)	22.9 ± 11.4	35.6 ± 34.1	0.528
*Surgical factors*			
Effective surgical time (min)	205.0 ± 52.2	168.5 ± 39.9	0.352
Tourniquet time (min)	146.0 ± 30.1	119.8 ± 28.4	0.268
Estimated blood loss (ml)	750.0 ± 390.5	358.0 ± 212.2	0.218
Extensive surgical approach (yes/no)	1/3	0/11	0.267
Hinged implant due to irreparable ligamentous instability (yes/no)	1/3	2/9	1.000
Significant bone defects requiring augments (yes/no)	4/0	10/1	1.000
Pathogen type			
G (+)	3	8	0.440
G (−)	1	0	
Fungus	0	2	
Mixed	0	1	
Antibiotics resistance (yes/no)	3/1	3/8	0.235
Sinus tract (yes/no)	1/3	2/9	1.000
Usage of transfusions	1/3	1/10	0.476
Total number of cement spacer insertion			
1	2	8	0.051
2	2	3	
3	0	0	
TKA type (primary/revision)	0/4	3/8	0.517
Prior DAIR surgery prior to cement spacer insertion	1/3	4/7	1.000
Time from initial surgery to cement spacer insertion (months)	29.3 ± 19.4	51.3 ± 52.4	0.255
Time from cement spacer insertion to prosthesis reimplantation (months)	2.4 ± 0.9	5.1 ± 2.9	**0.018**

BMI, body mass index; ASA, American Society of Anesthesiologists; PNI, prognostic nutritional index; CRP, C-reactive protein; ESR, erythrocyte sedimentation rate; WBC, white blood cell; G(+), Gram stain positive; G(−), Gram stain negative; TKA, total knee replacement arthroplasty; DAIR, debridement, antibiotics and implant retention. *p*-Value below 0.05 is written in bold text to note statistical significance

## Discussion

Through a survival analysis on PJI relapse of 65 knees that underwent two-stage reimplantation, the authors were able to identify three independent risk factors of PJI recurrence: (1) revision TKA at the time of initial PJI, (2) mixed pathogen-type infection, and (3) higher serum ESR level.

The recurrence rate of PJI of the knee after two-stage reimplantation in this study (23.1%) appears higher than the recurrence rates reported in some systematic reviews and meta-analyses. For example, Kunutsor et al. reported recurrence rates ranging from 8.8% to 16.2% in their systematic review and meta-analysis on the outcomes of one- and two-stage surgical revisions for infected knee prostheses [16]. Similarly, a recent meta-analysis by Goud et al. demonstrated reinfection rates of 9.4% for two-stage revisions of knee arthroplasties [17]. However, the recurrence rate in this study aligns more closely with findings from certain individual studies, such as a retrospective single-center study on 96 knees, which reported a recurrence rate of 18.8% [18]. Other studies have also documented variable outcomes: Pelt et al. reported a failure rate as high as 36% [19], while a single-center study on 20 knees documented a failure rate of 70% [8]. Despite these variations, two-stage revision remains the standard treatment for PJI of the knee in many institutions, as was performed in this study. Notably, some recent publications have suggested that single-stage and two-stage reimplantations may yield similar outcomes under specific circumstances [20].

Several studies have investigated the risk factors for relapse after two-stage reimplantation of PJI. A large multicenter study by Kheir et al. [21] identified ten significant risk factors for PJI treatment failure, including revision surgery and resistant organisms, both of which were also significant in our study. While Kheir et al. used different pathogen classifications, our finding of “mixed pathogen-type infection,” often involving G(−) bacteria, parallels their “resistant organisms” classification [22]. Interestingly, G(+) pathogens such as *Staphylococcus aureus* or resistant organisms such as MRSA were not associated with recurrence in our study. Chen et al. [23] identified male sex and positive intraoperative culture as risk factors for failure, and Hartman et al. [24] found elevated CRP and MSSA infections to predict higher reinfection rates, highlighting the role of inflammatory markers and specific pathogens, consistent with our findings of ESR level. Kubista et al. [25] reinforced the importance of monitoring high-risk patients after two-stage revision. Additionally, Sakellariou et al. [26] identified wound dehiscence and *Staphylococcus* carriers as risk factors for recurrence, while Lee et al. [27] noted male sex and positive cultures at reimplantation as predictors of early septic failure. While our study did not find associations with fungal infections or *Staphylococcus* carriers, our findings regarding mixed-pathogen infections align with broader pathogen-related risk factors for recurrence.

Although the risk factors identified in this study, such as type of TKA and pathogen type, have been reported in previous literature, our study is unique in applying survival analysis and Cox proportional hazard regression. This method, unlike previous studies, accounts for the time to infection recurrence, providing a time-dependent analysis of risk factors, which offers a more dynamic understanding of their impact on PJI recurrence.

While direct literature on infection recurrence rates in two-stage reimplantation for PJI in revision-type TKA is limited, Mortazavi et al. [11] provide relevant insights by demonstrating that patients undergoing revision TKA face a tenfold higher risk of infection compared with primary TKA. This elevated risk aligns with our finding of an alarming 80% recurrence rate (12 out of 15) in PJI cases after two-stage reimplantation for revision-type TKA, raising concerns about the effectiveness of current treatment methods. Given this high recurrence rate, there may be a need to revisit the standard approaches used for PJI management, particularly for revision-type TKAs. Potential strategies for improving outcomes could include extended antimicrobial therapies, changes in surgical protocols (e.g., longer interval between staged surgeries), or innovative techniques to enhance infection control. Further research is warranted to determine the most effective interventions for reducing the recurrence of infection in these high-risk patients.

A couple of recent studies have performed survival analysis and investigated the risk factors of PJI relapse after two-stage reimplantation. Kandel et al. [28] reported that liver disease (adjusted hazard ratio [aHR], 3.12; 95% confidence interval [CI], 2.09–4.66), the presence of a sinus tract (aHR, 1.53; 95% CI, 1.12–2.10), preceding debridement with prosthesis retention (aHR, 1.68; 95% CI, 1.13–2.51), a one-stage procedure (aHR, 1.72; 95% CI, 1.28–2.32), and infection due to Gram-negative bacilli (aHR, 1.35; 95% CI, 1.04–1.76) were significantly associated with the failure of PJI in hip and knee arthroplasties. Another study by Leitner et al. [29] reported lower age (*p* = 0.003), higher number of revisions before PJI (*p* = 0.007), and more than one microorganism at infection site (*p* = 0.034) as the risk factors of relapse. These findings were similar but had some differences with the results of our study. Contrary to these previous studies, patient-related factors, such as age and liver disease, were not significantly related to PJI recurrence in the current study.

As for the ancillary analysis on the type of pathogen at the time of PJI recurrence, limited information could be collected, because only 15 knees (4 with same-pathogen relapse versus 11 with different-pathogen relapse) were compared. Although it was not statistically significant, average time to recurrence was lower in the knees that recurred with the same type of pathogen. This finding was consistent with a previous study on pathogen type of recurred PJI by Garvin et al. [30].

This study has several limitations. First, as the study was retrospectively designed, it is vulnerable to selection bias, and therefore, future prospective studies are necessary for validation. Second, the diagnostic criteria and treatment protocols have changed over the study period (20 years) and may have affected the results of the study. However, as mentioned in the methods section, the treatment protocol of the study patients was grossly uniform in our institution over the study period, and thus, the authors believe that the limitation could be minimal. Third, the 44 variables that were analyzed in the study were mainly the previously established risk factors of PJI, and therefore, novel, unknown factors that may distinctively affect the recurrence of PJI after two-stage reimplantation may have been missed. Despite these limitations, three independent risk factors (revision TKA at the time of initial PJI, mixed pathogen-type infection, and higher serum ESR level) that negatively affect the outcome of two-stage reimplantation of PJI of the knee were identified in the study.

## Conclusions

A significant portion (23.1%) of prosthetic joint infection of the knees recurred after two-stage reimplantation. Revision TKA at the time of initial PJI, mixed pathogen-type infection, and higher serum ESR level were three significant risk factors of PJI recurrence. Surgeons should be more cautious in suspecting PJI relapse for these specific occasions.

## Data Availability

The datasets used and/or analyzed during the current study are available from the corresponding author on reasonable request.
